# Epidemic and Non-Epidemic Hot Spots of Malaria Transmission Occur in Indigenous *Comarcas* of Panama

**DOI:** 10.1371/journal.pntd.0004718

**Published:** 2016-05-16

**Authors:** William Lainhart, Larissa C. Dutari, Jose R. Rovira, Izis M. C. Sucupira, Marinete M. Póvoa, Jan E. Conn, Jose R. Loaiza

**Affiliations:** 1 Department of Biomedical Sciences, School of Public Health, State University of New York at Albany, Albany, New York, United States of America; 2 Wadsworth Center, New York State Department of Health, Albany, New York, United States of America; 3 Instituto de Investigaciones Científicas y Servicios de Alta Tecnología, Panama City, Panama; 4 Smithsonian Tropical Research Institute, Panama City, Panama; 5 Seção de Parasitologia, Instituto Evandro Chagas, Ananindeua, Pará, Brazil; Imperial College London, UNITED KINGDOM

## Abstract

From 2002–2005, Panama experienced a malaria epidemic that has been associated with El Niño Southern Oscillation weather patterns, decreased funding for malaria control, and landscape modification. Case numbers quickly decreased afterward, and Panama is now in the pre-elimination stage of malaria eradication. To achieve this new goal, the characterization of epidemiological risk factors, foci of transmission, and important anopheline vectors is needed. Of the 24,681 reported cases in these analyses (2000–2014), ~62% occurred in epidemic years and ~44% in indigenous *comarcas* (5.9% of Panama’s population). Sub-analyses comparing overall numbers of cases in epidemic and non-epidemic years identified females, *comarcas* and some 5-year age categories as those disproportionately affected by malaria during epidemic years. Annual parasites indices (APIs; number of cases per 1,000 persons) for *Plasmodium vivax* were higher in *comarcas* compared to provinces for all study years, though *P*. *falciparum* APIs were only higher in *comarcas* during epidemic years. Interestingly, two *comarcas* report increasing numbers of cases annually, despite national annual decreases. Inclusion of these *comarcas* within identified foci of malaria transmission confirmed their roles in continued transmission. Comparison of species distribution models for two important anophelines with *Plasmodium* case distribution suggest *An*. *albimanus* is the primary malaria vector in Panama, confirmed by identification of nine *P*. *vivax*-infected specimen pools. Future malaria eradication strategies in Panama should focus on indigenous *comarcas* and include both active surveillance for cases and comprehensive anopheline vector surveys.

## Author Summary

The study of malaria epidemiology and the spatial distributions of both malaria cases and vectors of malaria are essential for the elimination of this disease. Although Panama experienced a malaria epidemic in the early 2000s, this country reports fewer than 1,000 autochthonous cases each year. By understanding the risk factors for *Plasmodium* transmission and the locations of hot spots, vector and malaria control interventions can be targeted to both high risk individuals and regions, to maximize impact. In this research article, our results underscore the health disparities experienced by the indigenous people of Panama, as we identify them as those at greatest risk of malaria and their *comarcas* (indigenous reservations) as transmission hot spots. Additionally, we are able to implicate *Anopheles albimanus* as the primary vector of malaria in Panama through testing of collected specimens for *Plasmodium vivax* and *P*. *falciparum* infection and by calculating the odds of co-occurrence of both vectors with cases of malaria throughout Panama.

## Introduction

Between 2002 and 2005, Panama experienced a malaria epidemic which reached a peak of 5,085 cases in 2004 [1,2], associated with El Niño Southern Oscillation events [3], decreased malaria control funding [3,4] and extensive landscape modification/deforestation [5]. Intensified control efforts [6] quickly reduced the number of annually reported cases, with the Panamanian Ministry of Health (MINSA) reporting only 747 cases (100% *P*. *vivax*) in 2014, restricted to eastern Panama. Because of its current low level of malaria transmission, Panama is now in the pre-elimination stage of malaria eradication [7].

The process of malaria elimination will be difficult, as residual malaria cases are likely to occur in hard-to-access communities; thus the reported number of cases is an underestimate of the actual situation [8]. In 2014, ~63% of incident malaria cases in Panama occurred in the *comarcas* (indigenous reservations) of eastern Panama, near malaria endemic western Colombia [5]. Despite efforts to reduce migration of Colombians into Panama from this region, those successful at crossing the border are mobile, and rarely included in malaria surveillance programs [5]. Elimination-oriented control measures must identify spatial foci (hot spots) of residual infections using spatial tools, such as clustering analyses and risk mapping, to target interventions [9,10,11]. To increase the effectiveness of these control measures, it is useful to identify socio-demographic risk factors, using active surveillance with prompt treatment of identified cases (symptomatic and asymptomatic), evaluating current control methods by measuring their impact, and, finally, conducting vector biology studies to understand local anopheline ecology, biology and behavior [6].

Disparities exist between indigenous (Ngöbe-Buglé, Kuna Yala, Kuna de Madungandí, Kuna de Wargandí and Emberá-Wounaan) and non-indigenous Panamanians [12]. More than 96% of Panama’s indigenous population lives below the poverty line ($1,126 per person per year in 2008), compared to 17.7% among urban populations [3,12,13]. Additionally, indigenous Panamanians have a 7 to 9 year reduction in relative life expectancy [12]. A recent study has associated increased malaria incidence in Panama’s indigenous populations with living conditions, including homes built with temporary materials [11]. These underserved indigenous populations receive outpatient health services from MINSA, but access is reduced because of the prohibitively long and costly travel required to reach health posts [12].

Early research into Panama’s malaria vectors identified several *Plasmodium*-infected *Anopheles* species, including *Anopheles albimanus*, *An*. *pseudopunctipennis*, *An*. *tarsimaculata* (Syn. *An*. *aquasalis*), *An*. *bachmanni* (Syn. *An*. *triannulatus*), *An*. *neomaculipalpus*, *An*. *punctimacula* (Syn. *An*. *malefactor*), *An*. *argyritarsis*, *An*. *eiseni*, and *An*. *apicimacula s*.*l*. [14,15,16,17,18,19]. To date, 15 species and/or species complexes have been identified in Panama [2,20]. However, until recently, no single *Anopheles* species was formally incriminated as a vector of human *Plasmodium* spp. since the 1930s [2,20]. A 2015 study that tested anophelines from Comarca Kuna Yala (CKY) for *Plasmodium* infection found *An*. *albimanus* infected with *P*. *vivax* [21].

In 2008, Loaiza *et al*. [20] summarized selected vector biology metrics of anophelines collected over 35 years throughout the western Atlantic coast and eastern provinces/*comarcas* of Panama. These data provided important information on the complex nature of local *Plasmodium* transmission, and *An*. *albimanus* and *An*. *punctimacula s*.*l*. were identified as the most widespread and abundant anophelines [20]. *Anopheles albimanus* is a major regional malaria vector, with a distribution from southern Mexico to northern South America [22,23]. It is generally considered an exophagic, zoophilic vector that bites in the evening and throughout the night, though its behavior varies across its distribution [22,24]. *Anopheles punctimacula s*.*l*., a zoophilic vector [25], shares much of its distribution with *An*. *albimanus*, and has been observed from Mexico to Argentina, and in the Caribbean Islands [26,27].

The present study aims to address some of the knowledge gaps, advocated by [6], which might impede the implementation of effective malaria elimination strategies in Panama. The goal of elimination can be achieved by identifying risk factors associated with epidemic and non-epidemic malaria years in Panama, from 2000–2014, and determining the locations of malaria hot spots, both of which are critical during the pre-elimination stage of malaria eradication [11]. Additionally, this study aims to determine which *Anopheles* species are likely involved in malaria transmission, using spatial statistics, species distribution modeling, and testing of specimens for *Plasmodium* infection.

## Materials and Methods

### Malaria incidence, census, and geographic data

De-identified, national malaria incidence data (2000–2014) were obtained from the Department of Statistics and Vector Control of MINSA. These data included case location (*i*.*e*., province, district, and *corregimiento*–administrative subdivision of a district), age (years), and *Plasmodium* species (determined by blood smear microscopy). Cases identified as “imported” in the database were removed prior to analyses. Demographic information was obtained from the 2010 Panamanian census (Institute of Census and Statistics of the Comptroller office of the Republic of Panama) [28,29], and included both the total number of persons per *corregimiento* and the number of people in each province/*comarca* per 5-year age category. An ArcMap-compatible (ESRI, Redlands, California) shapefile depicting the geographic boundaries of Panama’s provinces, districts and *corregimientos* was obtained from the Smithsonian Tropical Research Institute (STRI) geographic information systems (GIS) information portal [30]. Case data that could not be matched to a *corregimiento* present in the STRI GIS shapefile were excluded prior to analyses.

### Epidemiological analyses

Chi-squared statistics were used to determine statistically significant differences in distributions of cases between non-epidemic (2000–2001, 2006–2014) and epidemic (2002–2005) years, by relevant demographic variables, including sex, age category (5-year intervals), location (*i*.*e*., province or *comarca*), and *Plasmodium* species. Logistic regression was used to determine statistically significant differences between non-epidemic and epidemic years (binary variable; 0 = non-epidemic, 1 = epidemic), while controlling for *Plasmodium* species (binary; 0 = *P*. *vivax*, 1 = *P*. *falciparum*) and multiple demographic variables simultaneously [province/*comarca* (binary; 0 = province, 1 = *comarca*), age in years (continuous), sex (binary; 0 = female, 1 = male), and an interaction variable between sex and province/*comarca*). Annual Parasite Indices (APIs; annual number of cases per 1000 persons) per *Plasmodium* species were plotted by year and location to visualize differences in temporal transmission intensities between provinces and *comarcas*. Finally, the average API in epidemic versus non-epidemic years was plotted versus age category, by sex and location, for each *Plasmodium* species separately, to better characterize risk factors for epidemic and non-epidemic malaria. Non-parametric Kolmogorov-Smirnov tests for equality in continuous distribution functions were employed to assess differences in age category patterns of cases by sex and *Plasmodium* species between epidemic and non-epidemic years. R v.3.1.3 software [31] and RStudio v.0.98.1091 (Boston, MA) were used for all statistical testing.

### Identification of spatial foci of increased transmission

Two cluster detection methods were used to identify malaria incidence hot spots for each year of the study: Kulldorff’s spatial scan statistic [32,33] and Getis-Ord Gi* [34,35]. The Kulldorff method was employed using Clusterseer software (BioMedware, Ann Arbor, Michigan) and requires spatial, census and case data. Getis-Ord statistics were undertaken using ArcMap software v.10.2.2 and the Spatial Statistics extension (Mapping Clusters >> Optimized Hot Spot Analysis), using spatial and incidence rate (API) information. All hot spot analyses were conducted using yearly case data or API information and the 2010 census. Additionally, the spatial location of cases was considered the centroid of the *corregimiento* from which the cases were reported. The number of centroids (*corregimientos*) per province and *comarca* can be found in Table 1. The results of the two spatial analyses were combined by summing the frequency of a *corregimiento* being identified (per year) by either or both methods. These frequency data were then summarized by summing the frequencies of each *corregimiento* in epidemic years and non-epidemic years separately, as in Xia *et al*. [36]. Maximum *corregimiento*-specific frequency in epidemic years is 8 (2 detection methods x 4 epidemic years), and the maximum in non-epidemic years is 22 (2 detection methods x 11 non-epidemic years). The summed frequencies were then projected onto the STRI GIS Panama shapefile to visualize their geographic locations and to provide a summarized distribution of the foci of increased malaria transmission during each period.

**Table 1:** Number of districts and *corregimientos* (centroids) per province or *comarca* in Panama.

Province	No. of Districts	No. of *Corregimientos*
Bocas del Toro	3	17
Chiriquí	13	96
Coclé	6	42
Colón	5	40
Darién	2	25
Herrera	7	48
Los Santos	7	80
Panamá	11	111
Veraguas	12	95
Comarca Emberá-Wounaan	2	5
Comarca Kuna Yala	1	4
Comarca Ngöbe-Buglé	7	58

### Species distribution modeling

Species distribution models for *An*. *albimanus* and *An*. *punctimacula s*.*l*. were generated using maximum entropy modeling, implemented in MaxEnt v.3.3.3k [37,38]. Species occurrence data were obtained from VectorMap [39] and through literature review [20,25,40,41,42,43,44,45,46,47,48,49,50,51,52,53,54,55,56,57], representing collection locations across the entire distribution of both species. MaxEnt models were built using climate and landscape variables, including 19 Bioclim variables (spatial resolution of 30 arc-seconds) [58], world soil suborder [59], altitude [60], hydrological variables (flow accumulation and flow direction) [61], and tree cover [62]. The MaxEnt program was run with default parameters, with the following differences: create response curves, jackknife to measure variable importance, random seed, do not write clamp grid when projecting, 25 random test percentage, 2 regularization multiplier, 15 replicates, subsample replicated run type, do not write output grids, and 5000 maximum iterations. A bias file was created for each species to inform the program that the area of interest was not sampled uniformly [63].

After running the full model for each species, predictor variables were assessed for their percent contribution to the model (≥ 3%) and pair-wise correlations (cut-off ≥|0.80|), as in Young *et al*. [64]. The percent contribution of each variable is given in the MaxEnt output. However, pairwise correlations were determined using the SDM Toolbox extension for ArcMap [65]. The predictor variables that met the above criteria were then used to create the final, parsimonious model, representing the average distributions of 15 MaxEnt iterations. These distributions, which represent the probability of species occurrence at each pixel of the map, were reclassified into a binary image (0 = absent, 1 = present) using the MaxEnt calculated maximum training sensitivity plus specificity logistic threshold in ArcMap. This threshold has been shown to be reliable when using presence-only data [66].

### Correlation of anopheline predicted distributions with incidence of malaria

Predicted *An*. *albimanus* and *An*. *punctimacula s*.*l*. distributions were compared, statistically, with those of malaria hot spots and API per *corregimiento* using odds ratios (99% confidence intervals), as in [67]. Eight comparisons were made per vector species (*P*. *vivax* and *P*. *falciparum* for API and hot spot analyses, separately, in epidemic versus non-epidemic years). Odds ratios (ORs) were calculated with the *raster* [68] and *abd* R packages [69], using binary presence/absence SDM maps compared to binary hot spot maps (0: never identified in a hot spot; 1: identified in a hot spot) or *corregimiento*-level API maps (0: unstable transmission, average API < 0.1 cases per 1,000; 1: stable transmission, average API ≥ 0.1 cases per 1,000).

### Collection and *Plasmodium* testing of anopheline specimens

**Fig 1:**
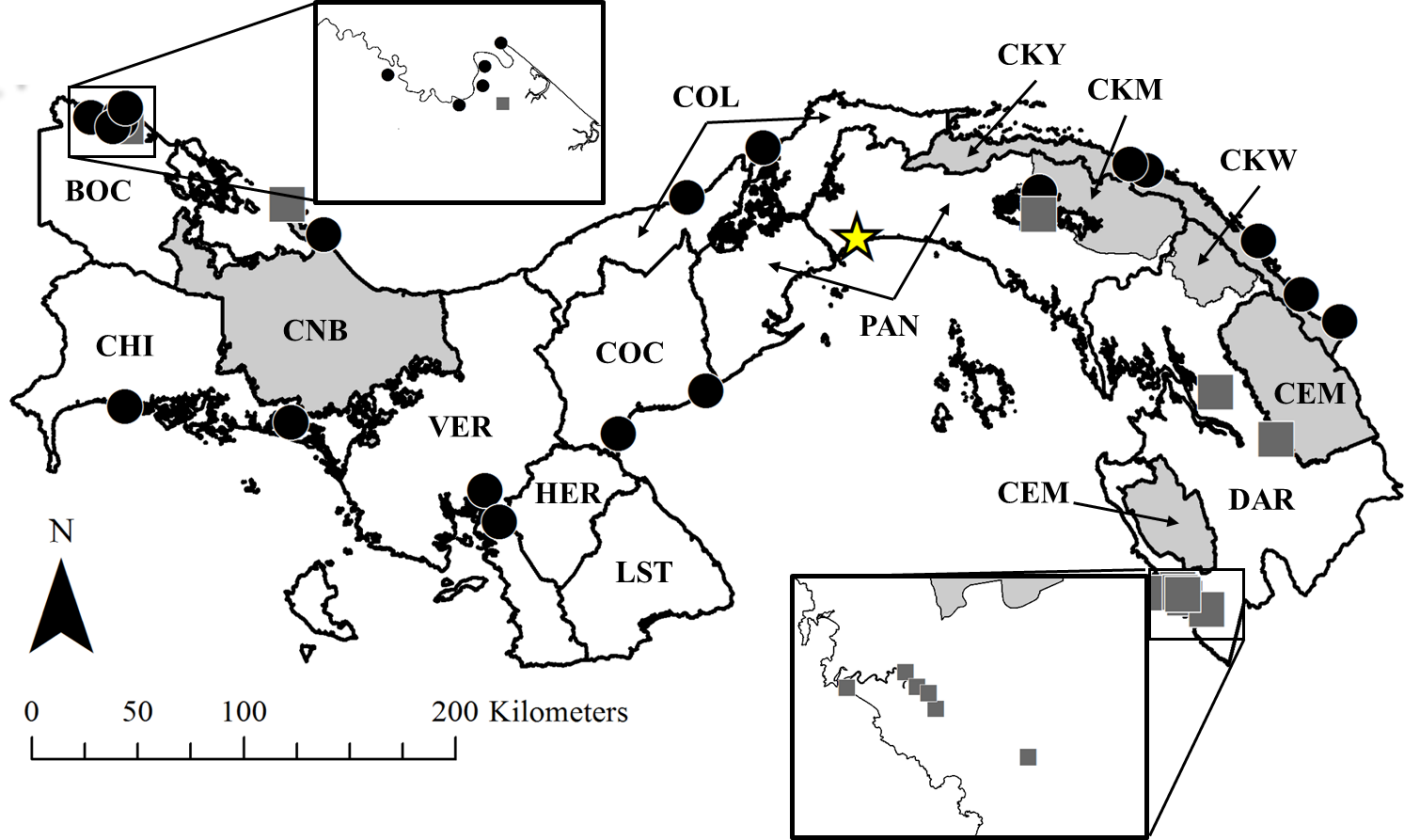
Map of Panama, depicting anopheline collection sites, provinces, and *comarcas*. 2006–2007 collection sites = black circles; 2008–2015 collection sites = grey squares; provinces = white; *comarcas* = grey. Yellow star indicates location of Panama City. Each province and *comarca* is labeled. BOC = Bocas del Toro; CHI = Chirquí, CNB = Comarca Ngöbe-Buglé, VER = Veraguas; HER = Herrera; LST = Los Santos; COC = Coclé, COL = Colón; PAN = Panamá, CKY = Comarca Kuna Yala; CKM = Comarca Kuna de Madungandí; CKW = Comarca Kuna de Wargandí, CEM = Comarca Embera-Wounaan; DAR = Darién. CKM is a territory within PAN province; CKW is a territory within DAR province. Insets depict details in northern BOC and in southwestern DAR provinces. Panama GIS shapefile obtained from STRI [30].

*Anopheles* mosquitoes were captured during overnight collections using human-landing catch, CDC light traps, Shannon traps, or resting. Collections occurred in thirty-one localities throughout Panama (n = 22 in 2006–2007, n = 14 in 2008–2015; Fig 1, S1 Table). Specimens were morphologically identified using a dichotomous key [70]. The heads and thoraces of specimens were pooled (n ≈ 5 per pool) by species, locality and collection date, and extracted using a Qiagen BioSprint 96 robot DNA extractor and Qiagen BioSprint 96 DNA Blood kits (Venlo, Netherlands). Specimens collected between 2006 and 2007 were tested for *Plasmodium* infection using ELISA [71], and those collected afterward (2008–2015) were tested using nested, real-time TaqMan PCR [72]. Inconclusive PCR results (*i*.*e*., *Plasmodium* genus-specific product amplified, but *P*. *vivax* and *P*. *falciparum*-specific products not amplified), were amplified again and the genus-specific product was sequenced and submitted to the National Center for Biotechnology Information (NCBI) Blastn database to assess species homology within the *Plasmodium* genus. Additionally, any *Plasmodium*-positive mosquito specimens not able to be morphologically identified (*e*.*g*., a member of a species complex) were molecularly identified using the ITS2 region of the mosquito 5.8S ribosomal RNA [73].

## Results

### Epidemiological data and analyses

**Fig 2:**
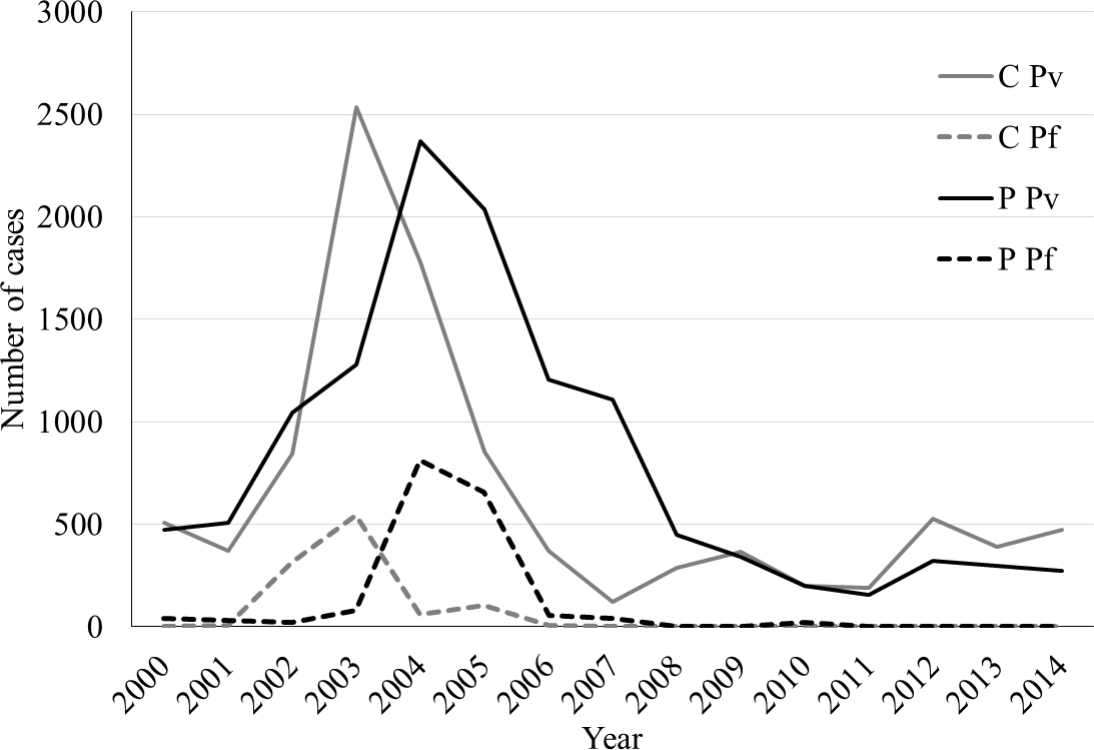
Number of cases of *Plasmodium vivax* and *Plasmodium falciparum* in the provinces and *comarcas* of Panama by year. Grey lines = *comarcas*; black lines = provinces; solid lines = *P*. *vivax*; dashed lines = *P*. *falciparum*.

Between 2000 and 2014, 24,937 malaria cases were reported from Panama. Of these, 256 (236 *P*. *vivax*, 16 *P*. *falciparum*, and 4 unknown) were excluded from analysis because the case *corregimiento* was not present in the Panama shapefile (n = 153), the age of the case was not available (n = 99), or the *Plasmodium* species was unknown (n = 4). The final data set included 24,681 cases, and no differences were noted in proportions of *Plasmodium* species between the original and final data sets. *Plasmodium vivax* cases were reported in every year of the study. However, no *P*. *falciparum* cases have been reported in Panama since 2010 (Fig 2). The epidemic peaks for both *P*. *vivax* and *P*. *falciparum* occurred in *comarcas* in 2003, and a year later in the provinces. During the malaria epidemic years (2002–2005), the average number of cases per year far exceeded that of non-epidemic years (2000–2001, 2006–2014) for both parasites (average 3.8-fold and 20.9-fold increases for *P*. *vivax* and *P*. *falciparum*, respectively). For all *P*. *falciparum* analyses, 2000–2001 and 2006–2010 were used as the non-epidemic years, since no *P*. *falciparum* cases were reported after 2010.

Chi-squared analyses detected statistically significant differences in the distribution of cases in epidemic and non-epidemic years by sex ($\chi_{df=1}^{2}$ = 4.968, *p* = 0.026), location ($\chi_{df=1}^{2}$ = 61.856, *p* < 0.001), age category ($\chi_{df=16}^{2}$ = 50.484, *p* < 0.001), and *Plasmodium* species ($\chi_{df=1}^{2}$ = 1228.476, *p* < 0.001; Table 2). During epidemic years, the indigenous people of the *comarcas*, females, and some age groups experienced more cases than expected. In addition, more cases of *P*. *falciparum* were reported in epidemic years than expected.

**Table 2:** Basic characteristics of malaria cases in Panama during non-epidemic and epidemic years (2000–2014).

Variable	Non-epidemic*		Epidemic^†^		X^2^ *p*-value
	no.	%	no.	%	
Sex					
Male	5506	58.93%	8816	57.48%	*p* = 0.026
Female	3838	41.07%	6521	42.52%	
Location					
Province	5536	59.25%	8301	54.12%	*p* < 0.001
*Comarca*	3808	40.75%	7036	45.88%	
Age Category					
0–4	1190	12.74%	1889	12.32%	*p* < 0.001^#^
5–9	1287	13.77%	2069	13.49%	
10–14	1150	12.31%	2144	13.98%	
15–19	1109	11.87%	1894	12.35%	
20–24	921	9.86%	1562	10.18%	
25–29	760	8.13%	1272	8.29%	
30–34	655	7.01%	1155	7.53%	
35–39	540	5.78%	806	5.26%	
40–44	509	5.45%	855	5.57%	
45–49	302	3.23%	390	2.54%	
50–54	270	2.89%	404	2.63%	
55–59	195	2.09%	274	1.79%	
60–64	186	1.99%	294	1.92%	
65–69	125	1.34%	159	1.04%	
70–74	82	0.88%	92	0.60%	
75–79	31	0.33%	41	0.27%	
80–84	25	0.27%	23	0.15%	
85–89	3	0.03%	9	0.06%	
90–94	2	0.02%	5	0.03%	
95–100	2	0.02%	0	0.00%	
Species					
*P*. *vivax*	9128	97.69%	12741	83.07%	*p* < 0.001
*P*. *falciparum*	216	2.31%	2596	16.93%	

* Non-epidemic years: 2000–2001, 2006–2014

† Epidemic years: 2002–2005

# Age categories 85–89, 90–94 and 95–100 were excluded from Chi-squared analyses due to low sample size.

Logistic regression analyses uncovered statistically significant differences between non-epidemic and epidemic years with respect to *Plasmodium* species and some demographic variables. The final, adjusted logistic model included *Plasmodium* species, province/*comarca*, age in years, sex, and an interaction term between sex and province/*comarca*. In epidemic years, there was a statistically significant 41% increase (odds ratio = 1.41, 95% confidence interval = 1.39–1.44) in the odds of a case being due to *P*. *falciparum* compared to non-epidemic years, controlling for all other variables. Additionally, in epidemic years, there was a statistically significant increase in the odds of a case occurring in a *comarca* (OR = 1.08, 95% CI = 1.06–1.10), compared to non-epidemic years, and controlling for all other variables. Finally, in epidemic years, there was a statistically significant interaction between sex and province/*comarca* resulting in a decrease in odds of a male case occurring in a *comarca* (OR = 0.97, 95% CI = 0.95–0.99), compared to non-epidemic years, and controlling for all other variables. Age (in years) and sex did not contribute to a statistically significant difference in odds of a case between non-epidemic and epidemic years in these analyses, after controlling for all other variables.

**Fig 3:**
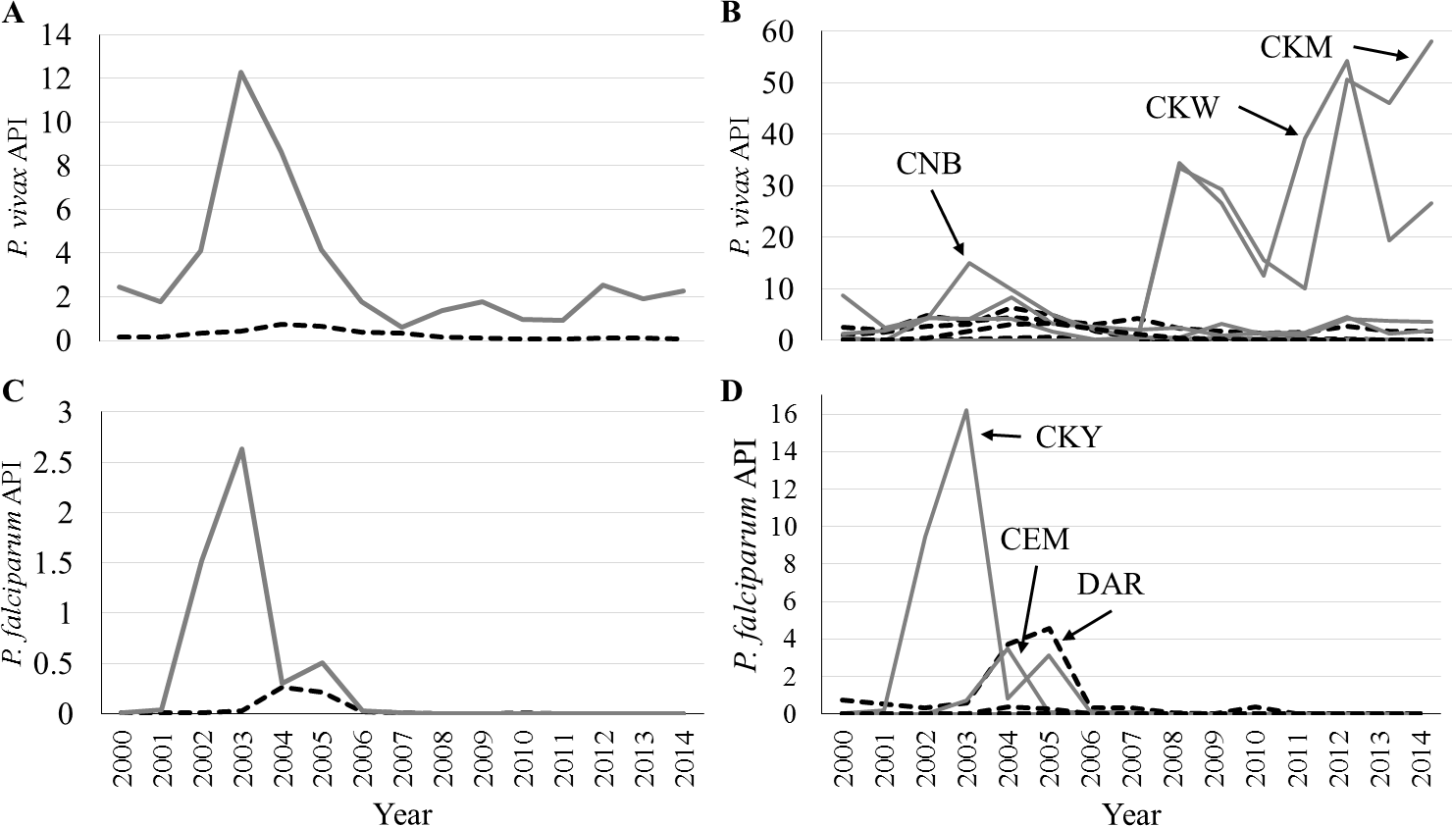
Annual Parasite Index (API) per year and location. Provinces = black dashed lines; *comarcas* = grey solid lines. **A** and **C**) *Plasmodium vivax* and *P*. *falciparum*, respectively, with provinces and *comarcas* grouped; **B** and **D**) *P*. *vivax* and *P*. *falciparum*, respectively, with provinces and *comarcas* separated. Note different y-axis scales on each panel. CNB = Comarca Ngöbe-Buglé, CKW = Comarca Kuna de Wargandí, CKM = Comarca Kuna de Madungandí, CKY = Comarca Kuna Yala, CEM = Comarca Emberá-Wounaan, DAR = Darién province.

Visualization of average API per *Plasmodium* species showed striking differences in malaria transmission and epidemiology experienced by provinces and *comarcas*. The API for *P*. *vivax* in *comarcas* was higher than that of the provinces for all years in this study (peak ~12.3 cases per 1,000 persons in 2003; Fig 3A). However, the *P*. *falciparum* API was higher only in the epidemic years (peak of ~2.6 cases per 1,000 persons in 2003) and nearly equal in non-epidemic years (Fig 3C). Further analysis by individual provinces and *comarcas* allowed for a more direct determination of the regions which had a disproportionate number of cases. CNB contributed the most *P*. *vivax* cases to the epidemic early on (~15 cases per 1,000 in 2003); in contrast, other *comarcas* and provinces, such as *Comarca* Emberá-Wounaan (CEM) and BOC reached their peaks in 2005 (Fig 3B). Despite their recognition as indigenous territories since January 1996 and July 2000 respectively [28], no cases of malaria were reported from Comarca Kuna de Madungandí (CKM) and Comarca Kuna de Wargandí (CKW) before 2008. However, since reporting began, the *P*. *vivax* API in both has increased, on average–a pattern unique to these *comarcas* (Fig 3B). Increases in *P*. *falciparum* API occurred primarily during the 2002–2005 malaria epidemic, beginning with a drastic increase in CKY (16.2 cases per 1,000 in 2003; Fig 3D). This increase was followed by subsequent increases in CEM (~3.5 cases per 1,000 in 2004), and in DAR (~3.7 and 4.6 cases per 1,000 in 2004 and 2005, respectively) and CKY (~3.1 cases per 1,000 in 2005) (Fig 3D).

**Fig 4:**
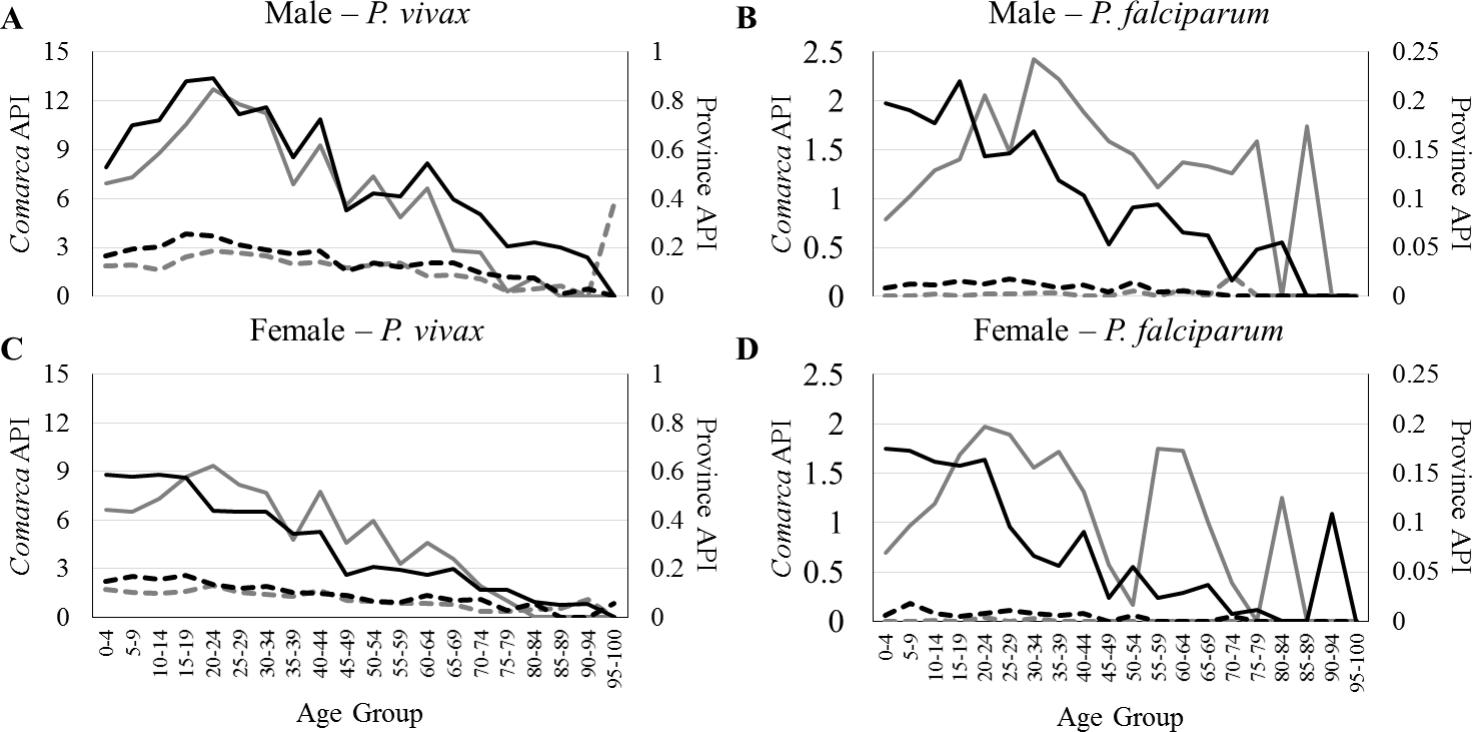
Annual Parasite Index (API) *versus* age group for epidemic and non-epidemic malaria years, by sex and location. Epidemic years = solid lines; non-epidemic years = dashed lines; provinces = black; *comarcas* = grey. Left y-axis represents *comarca* APIs and right y-axis represents province APIs. **A** and **C**) *Plasmodium vivax* API among males and females, respectively; **B** and **D**) *P*. *falciparum* API among males and females, respectively.

In general, average API per age category was greatest in *comarcas* in epidemic years for both sexes and both parasites (Fig 4). *Plasmodium vivax* API age category patterns (Fig 4A and 4C) do not differ greatly by province/*comarca* in epidemic and non-epidemic years, though male APIs were greater than those of females in general. Additionally, there is an overall decreasing trend in *P*. *vivax* API with increasing age (Fig 4A and 4C). However, *P*. *falciparum* APIs were much more variable across age categories (Fig 4B and 4D). Kolmogorov-Smirnov tests comparing age category-related patterns in cases between epidemic and non-epidemic years found no differences by sex and province/*comarca* for *P*. *vivax* (*comarca* males, *p* = 0.819; province males, *p* = 0.560; *comarca* females, *p* = 0.819; province females, *p* = 0.819). However, statistically significant differences in the age category-related patterns were identified for *P*. *falciparum* cases in all four categories (*p* < 0.001, *p* = 0.001, *p* < 0.001, and *p* = 0.348 respectively).

### Identification of spatial foci of increased transmission

**Fig 5:**
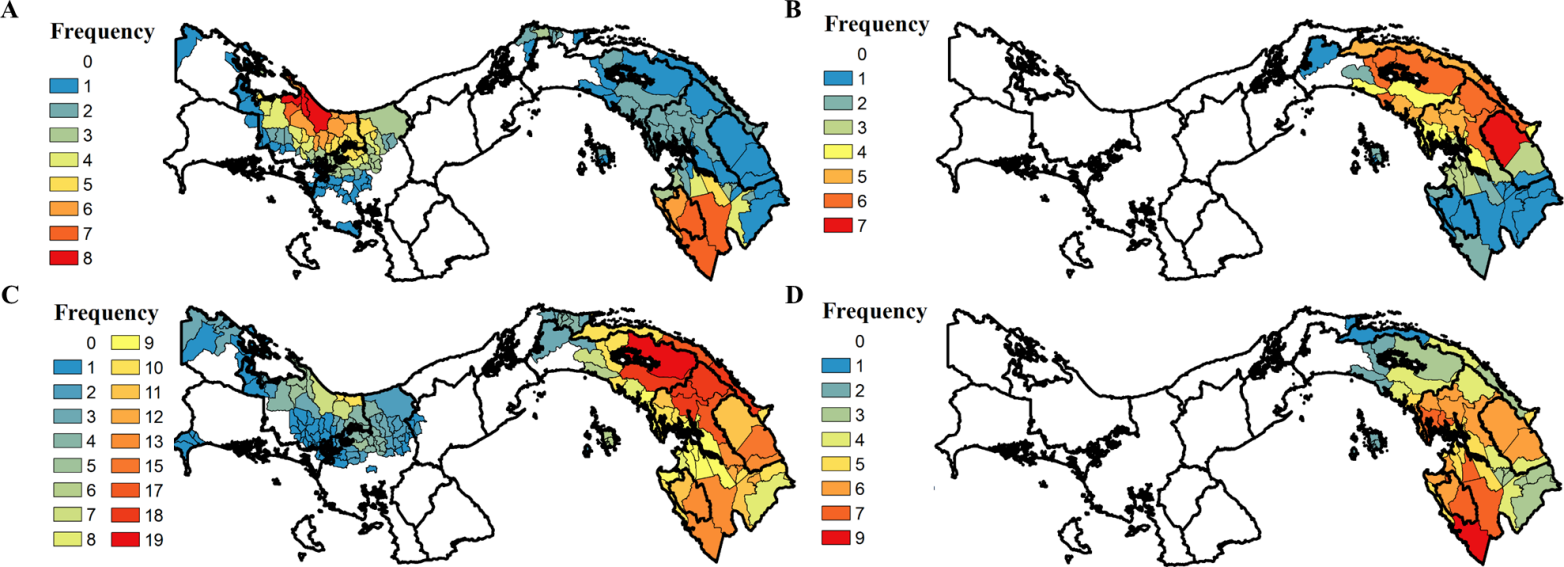
Total frequency of cluster occurrence for *Plasmodium vivax* and *P*. *falciparum* in Panama. *Corregimientos* are colored by the frequency at which they were identified by both hot spot detection methods over the designated period. **A**) *P*. *vivax* cluster frequency by *corregimiento* in epidemic years (2002–2005; maximum frequency = 8). **B**) *P*. *falciparum* cluster frequency by *corregimiento* in epidemic years (2002–2005; maximum frequency = 8). **C**) *P*. *vivax* cluster frequency by *corregimiento* in non-epidemic years (2000–2001, 2006–2014; maximum frequency = 22). **D**) *P*. *falciparum* cluster frequency by *corregimiento* in non-epidemic years (2000–2001, 2006–2010; maximum frequency = 14). Frequencies were calculated using data shown in S1 and S2 Figs. Panama GIS shapefile obtained from STRI [30].

Hot spot analyses show two major foci of increased *P*. *vivax* transmission in epidemic years: CNB, and southern DAR and the southwestern part of CEM (Figs 5A and S1). The locations of these foci differ from those of non-epidemic years, where only one major focus was observed in eastern Panama and included CKM, CKY, northern and southern DAR, CKW, and CEM (Figs 5C and S1). A smaller focus, identified less frequently in non-epidemic years, was observed in BOC, Veraguas (VER), and CNB (Fig 5C). Foci of *P*. *falciparum* transmission were only found in eastern Panama and largely overlapped between epidemic and non-epidemic years (Figs 5B, 5D and S2). The focus was centered near the Caribbean coast of eastern Panama in epidemic years and included CKM, CKY, northern DAR, CKW and northern CEM. However, it was centered near the Pacific coast in non-epidemic years, primarily in DAR and CEM.

### Species distribution models and correlation with *Plasmodium*

**Fig 6:**
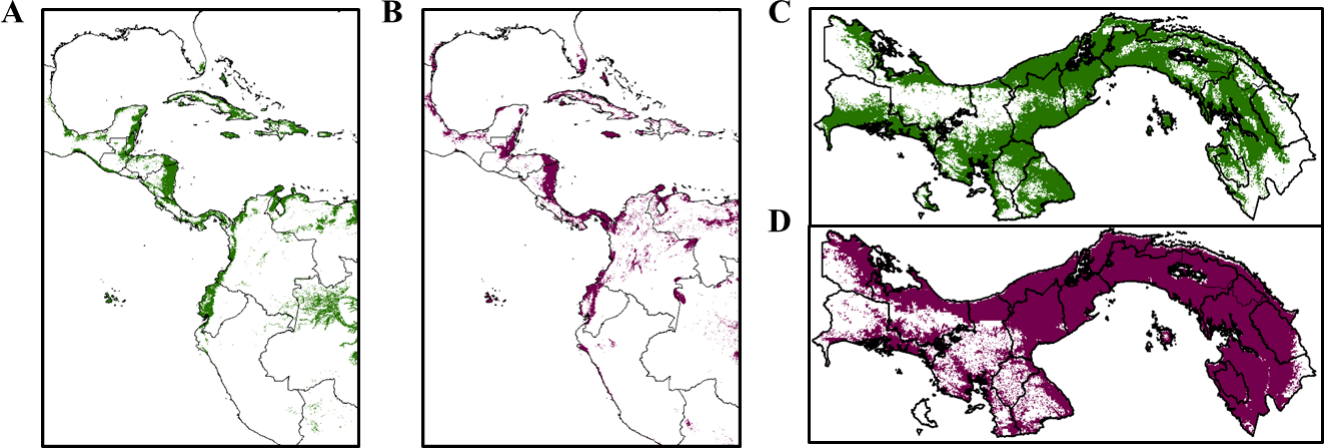
Species distribution models. **A** and **C**) *Anopheles albimanus* and **B** and **D**) *Anopheles punctimacula s*.*l*. in Panama. Panels **A** and **B** represent the full extent of the species distribution models. Panels **C** and **D** represent the distributions of each species within Panama. Color shading indicates areas of predicted suitable habitat/presence of the species; white indicates areas of predicted absence of the species. Central and South American GIS shapefiles freely available from DIVA-GIS [74]. Panama GIS shapefile obtained from STRI [30].

The *An*. *albimanus* full model produced a mean area under the curve (AUC) of 0.938. AUC ranges from 0.5 (random ranking of presence versus background sites) to 1.0 (perfect ranking), and is a value used to assess model performance [38]. Altitude, soil substrate, tree cover and Bioclim variables bio2 (mean diurnal range), bio4 (temperature seasonality), bio6 (minimum temperature of the coldest month), and bio9 (mean temperature of the driest quarter) all contributed ≥ 3% to the model. Pairwise variable correlations ≥ |0.8| were observed among the Bioclim variables bio2 and bio6 (bio6 was removed). The final/parsimonious model (mean AUC = 0.931) included altitude, soil substrate, tree cover, bio2, bio4, and bio9. The maximum training sensitivity plus specificity logistic threshold (0.1969) was used to create an *An*. *albimanus* species presence/absence species distribution map (Fig 6A).

The *An*. *punctimacula s*.*l*. full model produced a mean AUC of 0.885. Altitude, soil substrate, tree cover, bio2 (mean diurnal range), bio6 (minimum temperature of the coldest month), bio7 (annual temperature range), and bio14 (precipitation of the driest month) all contributed ≥ 3% to the model. Pairwise variable correlations ≥ |0.8| were observed among the Bioclim variables bio2, bio6 and bio7 (bio6 and bio7 were removed). The final/parsimonious model (mean AUC = 0.888) included altitude, soil substrate, tree cover, bio2, and bio14. The maximum training sensitivity plus specificity logistic threshold (0.2588) was used to create an *An*. *punctimacula s*.*l*. presence/absence species distribution map (Fig 6B).

Overall, *An*. *punctimacula s*.*l*. has a wider distribution than *An*. *albimanus* in Panama. Although areas of predicted *An*. *punctimacula s*.*l*. presence are found throughout western Panama and in nearly all areas of central and eastern Panama, areas of predicted *An*. *albimanus* presence tended to be found nearer to the coasts. In general, correlative analyses showed significantly increased odds of both *P*. *vivax* and *P*. *falciparum* (*corregimiento*-associated API and areas identified in hot spot analyses) in areas of predicted *An*. *albimanus* presence in both epidemic and non-epidemic years (Table 3). However, there was a significant reduction in odds of epidemic and non-epidemic *Plasmodium* transmission in areas of predicted *An*. *punctimacula s*.*l*. presence (Table 3).

**Table 3:** Comparison of predicted *Anopheles albimanus* and *An*. *punctimacula s*.*l*. distributions together with the distributions of *Plasmodium vivax* and *P*. *falciparum* cases in Panama.

			*An*. *albimanus*	*An*. *punctimacula s*.*l*.
*P*. *vivax*				
	API			
		Non-epidemic	1.68 (1.62–1.74)	0.36 (0.34–0.37)
		Epidemic	1.35 (1.30–1.40)	0.42 (0.40–0.43)
	Hotspot			
		Non-epidemic	2.30 (2.22–2.39)	0.36 (0.35–0.38)
		Epidemic	2.47 (2.38–2.57)	0.42 (0.40–0.44)
*P*. *falciparum*				
	API			
		Non-epidemic	1.02 (0.96–1.09)	0.04 (0.03–0.05)
		Epidemic	1.23 (1.18–1.28)	0.13 (0.12–0.14)
	Hotspot			
		Non-epidemic	1.13 (1.08–1.17)	0.10 (0.09–0.11)
		Epidemic	1.03 (0.99–1.07)	0.09 (0.08–0.10)

Values represent odds ratio (99% confidence interval)

API = annual parasite index

Hotspot = areas identified as part of a focus of increased malaria transmission by one or both detection methods

Non-epidemic = 2000–2001, 2006–2014 for *P*. *vivax*; 2000–2001, 2006–2010 for *P*. *falciparum*

Epidemic = 2002–2005 for both *Plasmodium* species

### *Plasmodium* testing of anophelines

Collecting efforts in thirty-one localities across Panama (Fig 1) resulted in 19,163 anopheline specimens (13,803 in 2006–2007 and 5,360 in 2008–2015; Table 4, S1 Table). Among these specimens, members of eight species (*An*. *albimanus*, *An*. *aquasalis*, *An*. *malefactor*, *An*. *neivai*, *An*. *neomaculipalpus*, *An*. *nuneztovari s*.*s*., *An*. *pseudopunctipennis*, and *An*. *vestitipennis*) and four species complexes (*An*. *apicimacula s*.*l*., *An*. *punctimacula s*.*l*., *An*. *strodei s*.*l*., and *An*. *triannulatus s*.*l*.; Table 4, S1 Table) were identified using morphological identification. Because the [70] morphological key is not reliable for all anopheline species in eastern Panama, specimens originally considered to be *An*. *oswaldoi* were molecularly identified using the *COI* barcode, as in [75]; all these specimens were *An*. *nuneztovari s*.*s*. Four specimens could not be identified morphologically (2 –*Anopheles* (*Nyssorhynchus*) spp. and 2 –*Anopheles* (Arribalzagia series) spp.).

**Table 4:** *Anopheles* mosquito specimens collected throughout Panama, for *Plasmodium* testing (Fig 1).

Province	*Anopheles* species	2006–2007		2008–2015	
		# Tested	# Infected (ELISA)	# Tested	# Infected (rtPCR)
BOC	*albimanus*	5990	9	120	
BOC	*aquasalis*	3			
BOC	*neomaculipalpus*	186			
BOC	*punctimacula s*.*l*.	340		60	1
BOC	*strodei s*.*l*.	24		320	
BOC	*vestitipennis*	210			
CHI	*albimanus*	560			
CKM	*albimanus*	405			
CKM	*punctimacula s*.*l*.	15			
CKM	*triannulatus s*.*l*.	51			
CKY	*albimanus*	1020		291	
CKY	*apicimacula s*.*l*.	12			
CKY	*aquasalis*	135		146	
CKY	*malefactor*	9			
CKY	*punctimacula s*.*l*.	65		72	
CKY	*pseudopuctipennis*	30			
CKY	*strodei s*.*l*.			84	
CNB	*albimanus*	2405		331	
CNB	*apicimacula s*.*l*.			781	
CNB	*aquasalis*	9		78	
CNB	*neivai*	63		152	
CNB	*neomaculipalpus*			5	
CNB	*punctimacula s*.*l*.	735			
COC	*albimanus*	200			
COL	*albimanus*	520			
COL	*aquasalis*	69			
DAR	*albimanus*	90		1017	
DAR	*Anopheles* (Arribalzagia series) sp.			2	
DAR	*Anopheles* (*Nyssorhynchus*)			2	
DAR	*apicimacula s*.*l*.			125	
DAR	*malefactor*			7	
DAR	*neomaculipalpus*			9	
DAR	*nuneztovari s*.*s*.			754	
DAR	*pseudopuctipennis*			38	
DAR	*punctimacula s*.*l*.	257		786	
DAR	*strodei s*.*l*.			82	
DAR	*triannulatus s*.*l*.			53	
PAN	*punctimacula s*.*l*.			5	
PAN	*strodei s*.*l*.			40	
VER	*albimanus*	400			

ELISA = Enzyme-Linked ImmunoSorbent Assay, rtPCR = real-time Polymerase Chain Reaction, BOC = Bocas del Toro, CHI = Chiriquí, CKM = Comarca Kuna de Madungandí, CKY = Comarca Kuna Yala, CNB = Comarca Ngöbe-Buglé, COC = Coclé, COL = Colón, DAR = Darién, PAN = Panamá, VER = Veraguas. Additional collection information can be found in S1 Table.

ELISA testing of specimens collected in 2006–2007 identified nine pools of *An*. *albimanus* infected with *P*. *vivax* (3 with VK210 variant and 6 with VK247 variant). These specimens were collected in BOC (Fig 1, Table 4, S1 Table). Real-time PCR of specimen pools from 2008–2015 resulted in the identification of one *Plasmodium* spp. positive *An*. *punctimacula s*.*l*. pool (resting collection in BOC, Table 4, S1 Table). This PCR result was inconclusive, and the *Plasmodium* genus-specific PCR product sequence analysis showed the presence of *Plasmodium juxtanucleare* (163 bp fragment of 18S ribosomal RNA gene, 99% identity, 0 gaps, GenBank accession AF463507.1). Further analysis of the *Plasmodium* positive *An*. *punctimacula s*.*l*., using ITS2, determined that it belongs to clade B (100% identity, 0 gaps, GenBank accession JX212812.1). Furthermore, the collection site of this mosquito pool is congruent with the published distribution of *An*. *punctimacula* clade B in Panama [76].

## Discussion

As Panama makes progress toward malaria elimination, a greater push for identification of cases in the remote *corregimientos* and *comarcas* of the country will be essential. Despite increases in the number of cases among some demographic groups, such as females, during epidemic years, the indigenous people of Panama were identified as those most disproportionately affected by malaria in all years of this study (Table 2, Figs 3 and 4). Many indigenous people live in poverty and have limited access to health services [3,12]. In 2014, with only 747 *P*. *vivax* and 0 *P*. *falciparum* cases nationally, the people living within the indigenous territories of CKM and CKW experienced APIs of ~58 and 27 cases per 1,000 people, respectively (Fig 3). The province or *comarca* with the third highest API in 2014 in Panama (CKY), had an API of ~3.6 (17-fold and 7-fold lower than CKM and CKW). Even though these territories have relatively small populations (CKM: 4,271; CKW: 1,914) [28], they serve as an important focus of residual malaria transmission.

During the 2002–2005 malaria epidemic, there was a marked increase in the number of *P*. *falciparum* cases (16.9% of cases in epidemic years, 2.3% of cases in non-epidemic years; Table 2, Fig 2). This *P*. *falciparum* epidemic was restricted to eastern Panama, and primarily affected CKY, CEM and DAR (Figs 3 and 5). Restriction of cases near the Colombian border is consistent with the results of a recent study characterizing *P*. *falciparum* haplotypes in Panama, confirming Colombia as their origin [77]. However, the *P*. *vivax* epidemic was more widespread, affecting eastern Panama, along with CNB and BOC in the west (Figs 3 and 5). Interestingly, only one major non-epidemic focus of *P*. *vivax* was identified, and it was centered in eastern Panama (Figs 3 and 5). These results suggest that current *P*. *vivax* transmission in Panama could be related to the influx and movement of migrants from the malarious regions of western Colombia [5].

For the most part, Kulldorff’s spatial scan statistic and Getis-Ord Gi* provided congruent results in analyses identifying spatial foci of increased *Plasmodium* transmission (S1 and S2 Figs). However, there are some instances where small numbers of cases in a given region were identified by only one method as a hot spot. For example, hot spots were identified by Kulldorff’s scan statistic for *P*. *vivax* (S1 Fig, black circles; 2003, 2005, 2006, 2008–2014) and *P*. *falciparum* (S2 Fig, black circles; 2010), despite not being identified by Getis-Ord Gi*. The opposite is true, too. Getis-Ord Gi* identified areas of statistically significantly increased incidence of *P*. *vivax* (S1 Fig, pink/red shading; 2005) and in *P*. *falciparum* (S2 Fig, pink/red shading; 2009). These findings support the use of two separate statistical methods for the identification of hot spots of *Plasmodium* incidence, and the combination of the results from the two methods allows for a better, more complete picture of the true spatial heterogeneity of malaria cases in Panama over time.

With the exception of *P*. *falciparum* in *comarcas* during epidemic years, *Plasmodium* APIs tended to decrease with increasing human age, suggesting that some individuals in Panama, especially in *comarcas*, could be asymptomatic carriers/reservoirs of the parasite [78,79]. Similar patterns have been reported elsewhere [78,80,81,82,83], and, in some cases, asymptomatic patients have been shown to outnumber symptomatic ones by 4–5 fold [80]. Determining whether there are asymptomatic carriers of *Plasmodium* among those living in areas with continued malaria transmission in Panama is essential for current and future malaria elimination efforts. These individuals cannot be identified through passive disease surveillance, but must be detected through active case detection strategies with PCR testing of samples, as suggested by [84], and must be accompanied by comprehensive treatment.

In this study, we identified nine specimen pools of *An*. *albimanus* infected with *P*. *vivax*. However, because collection efforts were spread throughout Panama and, in general, of short duration, it is possible that other important local vectors may have been missed. However, the absence of *Plasmodium* infected anopheline pools from 2008–2015 may truly reflect the low level of malaria endemicity in Panama, though a recent study in CKY found the presence of *P*. *vivax*-infected *An*. *albimanus* in CKY [21]. *Anopheles* mosquito surveys and *Plasmodium* testing should be repeated using the epidemiological and spatial statistics results presented here as a framework for locality identification. For example, these surveys should be completed in the indigenous *comarcas* (*e*.*g*., CKM and CKW) since indigenous people reported 63.2% of the malaria cases in Panama during this study, but make up only 6.2% of the population, according to the 2010 census [28]. Also, mosquito-sampling effort should increase in future studies to account for the potential regional transmission role of *An*. *darlingi*, especially in southern DAR.

*Anopheles albimanus* was identified in this study as the most important malaria vector in Panama. However, the lack of an association between the distributions of *An*. *punctimacula s*.*l*. and *Plasmodium* does not mean that this species has no role in local transmission. Within this species complex, there are multiple molecular lineages [76], and further research is needed to model their individual distributions and to characterize the possible importance of each in malaria transmission. One of the assumptions of the spatial hot spot analyses in this study is that incident malaria occurred randomly throughout each *corregimiento* since it was not possible to georeference every locality with reported malaria cases. *Anopheles albimanus* has increased odds of co-occurrence and is likely the major vector in this country, but this does not mean that *An*. *punctimacula* and other anophelines did not play important roles in local transmission of *Plasmodium* in both epidemic and non-epidemic years. Additionally, because *An*. *punctimacula* appears to be much more of an ecological generalist, compared to *An*. *albimanus* in these analyses, it is predicted to be present in many areas where no malaria transmission was reported during the study period, greatly diminishing its odds of co-occurrence with incident malaria and potentially underestimating its importance as a vector in Panama. Among the anophelines tested for *Plasmodium*, one pool of *An*. *punctimacula s*.*l*. (collected resting in BOC) was positive for *Plasmodium juxtanucleare*, an avian parasite [85,86,87,88]. Despite being found in an anopheline (previous work suggests that *Anopheles* mosquitoes are refractory to this parasite [89]), the primary vectors of *P*. *juxtanucleare* are normally *Culex* spp. [89,90,91,92].

This study has a number of limitations. Firstly, the epidemiological analyses used in this study relied on malaria cases reported to the Panamanian MINSA either passively, through patients visiting health posts, or actively by MINSA workers visiting villages with current malaria cases. Despite the hard work of the Panamanian MINSA, the number of reported malaria cases in Panama, particularly those within *comarcas*, is likely to be an underestimate, due to the difficulty accessing remote villages and/or traveling from these villages to local health posts. Secondly, some administrative areas in Panama underwent restructuring between the 2000 and 2010 censuses. Because of these changes, and because the GIS shapefiles used in this study reflected the current administrative boundaries in Panama, it was not possible to use the 2000 census data for API calculations. As a result, we used 2010 census numbers for all years of the study, knowing that these numbers may not reflect the true populations in each province, district or municipality during all years. Thirdly, because it was not possible to georeference each reported malaria case, data were aggregated to summarize each *corregimiento* per year, reducing the resolution of the spatial hot spot analyses. Finally, despite the mosquito collections summarized in this study occurring during times of known malaria transmission, these collections occurred after the 2002–2005 epidemic, making it impossible to determine the vectors playing roles in transmission during this period. However, Loaiza *et al*. [20] summarizes 35 years of anopheline collections in Panama, and throughout that time period, *An*. *albimanus* was found to be the most abundant vector, suggesting that it was likely to play an important role in 2002–2005 malaria epidemic in Panama.

Recent research on malaria hotspots in areas of low or unstable transmission has shown the importance of PCR and serology based data for identification of regions with increased levels of asymptomatic carriage of *Plasmodium* parasites [93,94,95], rather than the use of microscopy [79,93]. In Panama, we recommend the use of PCR for the identification of people with low levels of parasitemia, rather than serology, because high antibody titers may not represent a current infection, but rather a past exposure [96]. After prospective surveys of the hot spots identified in this study, using PCR identification of *Plasmodium* infection, these data can be analyzed using Bayesian geostatistics [97] to predict the spatiotemporal patterns of asymptomatic *Plasmodium* infections in Panama, giving further guidance to malaria elimination efforts. A recent cluster-randomized, controlled trial tested the effectiveness of interventions targeting malaria hot spots and identified important factors that need to be recognized and addressed in the future [98]. Bousema *et al*. [98] suggest the failure of their interventions to decrease local malaria transmission is due to unrecognized insecticide resistance in the local vector populations, transmission of *Plasmodium* or spread of infected mosquitoes from other, local transmission hot spots, and/or introduction of *Plasmodium* through human movement/migration.

Overall, this study underscores the disparities between the indigenous and non-indigenous people of Panama, with respect to health care access. Current and future malaria elimination efforts must be focused on the *comarcas* to maximize their effect, and must include active surveillance systems to identify asymptomatic reservoirs of *Plasmodium*, and thorough anopheline surveys, to identify the species that are involved in transmission. Vectors identified as important in the identified hot spots should be studied to determine their feeding and resting behaviors, biting time patterns, population genetics and insecticide resistance, in order to create tailored, effective vector control interventions. Additionally, human movement and migration must be better studied and understood in Panama, since it is likely that *Plasmodium* transmission in eastern Panama is associated with the immigration of people from Colombia, and because the importation of *Plasmodium* is known to hinder malaria elimination programs [99,100].

## Supporting Information

S1 Table *Anopheles* specimen collection and *Plasmodium* infection information. (PDF)

S1 Fig Identification of annual foci of increased *Plasmodium vivax* malaria incidence in Panama (2000–2014) using two detection methods. Colored municipalities (Getis-Ord Gi* statistic; red and pink = 99% and 95% confidence, respectively) and circled areas (Kulldorff’s spatial scan statistic) signify statistically significant malaria incidence hot spots. Dark (99% confidence) and light blue (95% confidence) colored municipalities in the 2005 panel represent cold spots of malaria incidence, as determined by Getis-Ord Gi* statistics.
(TIFF)

S2 Fig Identification of annual foci of increased *Plasmodium falciparum* malaria incidence in Panama (2000–2014) using two detection methods. No foci were identified for *P*. *falciparum* for years 2011–2014. Colored municipalities (Getis-Ord Gi* statistic; red and pink = 99% and 95% confidence, respectively) and circled areas (Kulldorff’s spatial scan statistic) signify statistically significant malaria incidence hot spots. No statistically significant hot spot was detected by Kulldorff’s spatial scan statistics in 2009, whereas none were detected by Getis-Ord Gi* statistics in 2010.
(TIFF)
