# Supplementary material for: Epidemic and Non-Epidemic Hot Spots of Malaria Transmission Occur in Indigenous Comarcas of Panama
Source: PLoS Negl Trop Dis. 2016 May 16;10(5):e0004718. doi: 10.1371/journal.pntd.0004718 (PMC4868294; doi:10.1371/journal.pntd.0004718)
Supplement: S1 Table — (PDF) [file pntd.0004718.s001.pdf]

S1 Table. ***Anopheles*** **specimen collection and** ***Plasmodium*** **infection information.**

Province	Locality	Latitude	Longitude	*Anopheles* species	Collections			
					2006 - 2007		2008 - 2015	
					# Collected	# Positive[a]	# Collected	# Positive[b]
BOC	Barranco Montana	9.53049	-82.71550	*albimanus*	870	2[c]		
				neomaculipalpus	6			
	Finca 51	9.49300	-82.56600	*albimanus*			120	
				punctimacula s.l.			60	1[d]
				strodei s.l.			320	
	Finca California	9.54163	-82.58951	*albimanus*	1535	3[e]		
				neomaculipalpus	6			
				vestitipennis	27			
	Finca Debora	9.51639	-82.59212	*albimanus*	930			
	Puente Blanco	9.49121	-82.62281	*albimanus*	1905	4[f]		
				neomaculipalpus	174			
				punctimacula s.l.	330			
				strodei s.l.	24			
				vestitipennis	174			
	Sixaola	9.57211	-82.56854	*albimanus*	750			
				aquasalis	3			
				punctimacula s.l.	10			
				vestitipennis	9			
CHI	La Barqueta	8.30603	-82.57309	*albimanus*	420			
	Las Lajas	8.23991	-81.86772	*albimanus*	140			
CKM	Canaza Bayano	9.21732	-78.69158	*albimanus*	405			
				punctimacula s.l.	15			
				triannulatus s.l.	51			
CKY	Carreto	8.78201	-77.58046	*albimanus*	5			
				apicimacula s.l.	6			
				aquasalis	57			
				malefactor	3			
				punctimacula s.l.	10			
	Isla Pino	9.00764	-77.76249	*albimanus*	245			
				aquasalis	18			
				punctimacula s.l.	5			
				pseudopunctipennis	6			
	Playon Chico	9.30794	-78.23572	*albimanus*	10		161	
				apicimacula s.l.	6			
				aquasalis	51		131	
				pseudopunctipennis	24			
				malefactor	6		17	
				punctimacula s.l.	10			
	Puerto Obaldia	8.66530	-77.41690	*albimanus*	60		130	
				aquasalis			15	
				punctimacula s.l.			55	
				strodei s.l.			84	
	Ukupa	9.33450	-78.30775	*albimanus*	700			
				aquasalis	9			
				punctimacula s.l.	40			
CNB	Hilo Creek	9.16500	-81.88300	*albimanus*	30		3	
				apicimacula s.l.			697	
				aquasalis	9		78	
				neivai	63		152	
				punctimacula s.l.	285			
	Rio Diablo	9.03484	-81.72854	*albimanus*	2375		328	
				apicimacula s.l.			84	
				neomaculipalpus			5	
				punctimacula s.l.	450			
COC	El Salao	8.19198	-80.47848	*albimanus*	45			
	Santa Clara	8.37358	-80.10796	*albimanus*	155			

S1 Table continued

Province	Locality	Latitude	Longitude	*Anopheles* species	Collections 2006 - 2007 # Collected	Collections 2006 - 2007 # Positive[a]	Collections 2008 - 2015 # Collected	Collections 2008 - 2015 # Positive[b]
COL	Galeta	9.40341	-79.86389	*aquasalis*	69			
	Rio Indio	9.19128	-80.18601	*albimanus*	520			
DAR	Aruza Abajo	8.36900	-77.94200	*albimanus*			21	
				Anopheles (Arribalzagia series)			2	
				Anopheles (*Nyssorhynchus*)			2	
				malefactor			7	
				neomaculipalpus			9	
				pseudopunctipennis			8	
				punctimacula s.l.			6	
				triannulatus s.l.			36	
	Biroquera	7.53478	-78.10464	*albimanus*			430	
				apicimacula s.l.			80	
				punctimacula s.l.			270	
	El Coco	7.49747	-78.07400	*albimanus*			30	
				apicimacula s.l.			15	
				nuneztovari s.s.			225	
				punctimacula s.l.			90	
	Jaque	7.51875	-78.16392	*albimanus*			146	
				punctimacula s.l.			35	
				strodei s.l.			20	
				triannulatus s.l.			17	
	La Penita	7.44833	-77.98044	*albimanus*			184	
				pseudopunctipennis			10	
				punctimacula s.l.			20	
	Pavarando	7.51975	-78.09305	*albimanus*			52	
				nuneztovari s.s.			135	
				pseudopunctipennis			20	
				punctimacula s.l.			30	
	Valle Alegre	7.51333	-78.08131	*albimanus*			14	
				nuneztovari s.s.			394	
				punctimacula s.l.			65	
	Yaviza	8.16972	-77.68722	*albimanus*	90		140	
				apicimacula s.l.			30	
				punctimacula s.l.	257		270	
				strodei s.l.			62	
PAN	Aguas Claras	9.12389	-78.69250	*punctimacula s.l.*			5	
				strodei s.l.			40	
VER	El Bongo	7.95163	-81.04417	*albimanus*	10			
	Las Colomas	7.81675	-80.98401	*albimanus*	390			

BOC = Bocas del Toro; CHI = Chiriquí; CKM = Comarca Kuna de Madungandí; CKY = Comarca Kuna Yala; CNB = Comarca Ngöbe-Buglé; COC = Coclé; COL = Colón; DAR = Darién; Pan = Panamá; VER = Veraguas. [a] Specimens collected from 2006 - 2007 were tested for *Plasmodium* infection using ELISA [71]. [b] Specimens collected from 2008 - 2015 were tested for *Plasmodium* infection using real-time PCR [72]. [c] Two *Anopheles albimanus* pools found infected with *Plasmodium vivax* variant VK247. [d] One *Anopheles punctimacula* specimen found infected with *Plasmodium juxtanucleare.* [e] Three *Anopheles albimanus* pools found infected with *Plasmodium vivax* variant VK247. [f] Three *Anopheles albimanus* pools found infected with *Plasmodium vivax* variant VK210, and one pool found infected with VK247.
