# Supplementary figures and images for: Epidemic and Non-Epidemic Hot Spots of Malaria Transmission Occur in Indigenous Comarcas of Panama
Source: PLoS Negl Trop Dis. 2016 May 16;10(5):e0004718. doi: 10.1371/journal.pntd.0004718 (PMC4868294; doi:10.1371/journal.pntd.0004718)

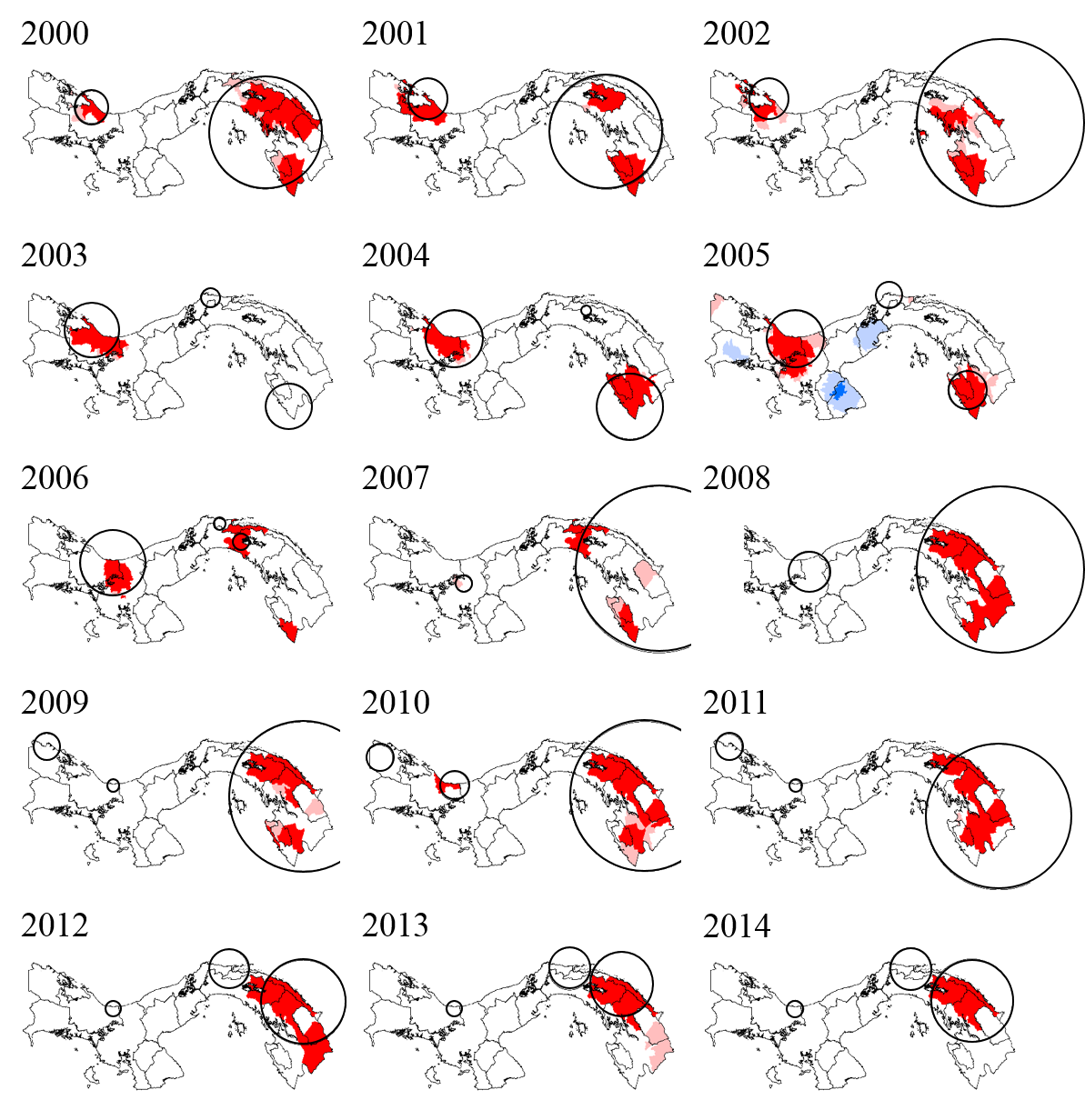

Supplement: S1 Fig — Colored municipalities (Getis-Ord Gi* statistic; red and pink = 99% and 95% confidence, respectively) and circled areas (Kulldorff’s spatial scan statistic) signify statistically significant malaria incidence hot spots. Dark (99% confidence) and light blue (95% confidence) colored municipalities in the 2005 panel represent cold spots of malaria incidence, as determined by Getis-Ord Gi* statistics. (TIFF) [file pntd.0004718.s002.tiff]

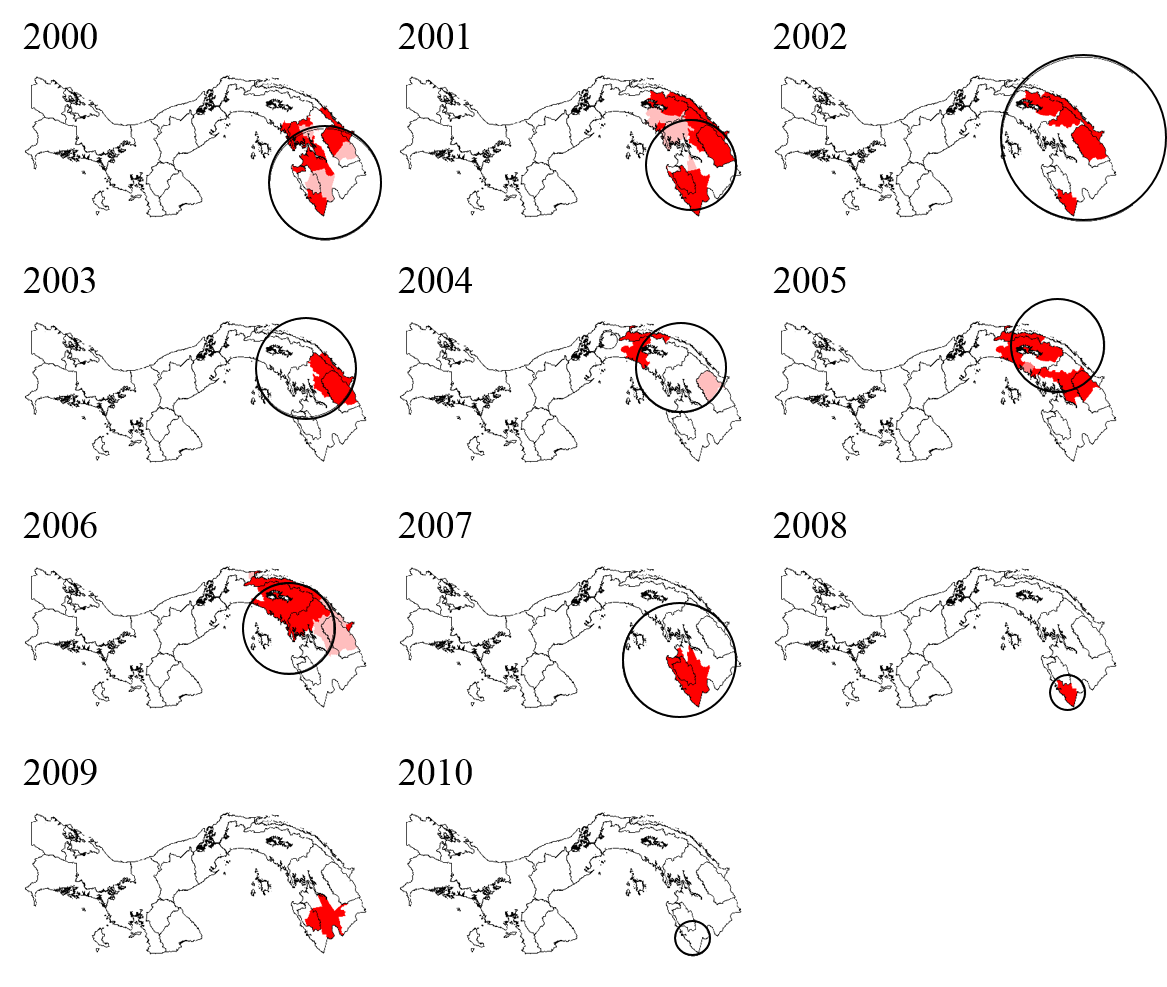

Supplement: S2 Fig — No foci were identified for P. falciparum for years 2011–2014. Colored municipalities (Getis-Ord Gi* statistic; red and pink = 99% and 95% confidence, respectively) and circled areas (Kulldorff’s spatial scan statistic) signify statistically significant malaria incidence hot spots. No statistically significant hot spot was detected by Kulldorff’s spatial scan statistics in 2009, whereas none were detected by Getis-Ord Gi* statistics in 2010. (TIFF) [file pntd.0004718.s003.tiff]
